# Supplementary material for: Mobile ear-EEG to study auditory attention in everyday life: Auditory attention in everyday life
Source: Behav Res Methods. 2021 Mar 15;53(5):2025–36. doi: 10.3758/s13428-021-01538-0 (PMC8516794; doi:10.3758/s13428-021-01538-0)
Supplement: Supplementary file 1 — (PDF 579 KB) [file 13428_2021_1538_MOESM1_ESM.pdf]

Supplementary material to

'Mobile ear-EEG to study auditory attention in everyday life'

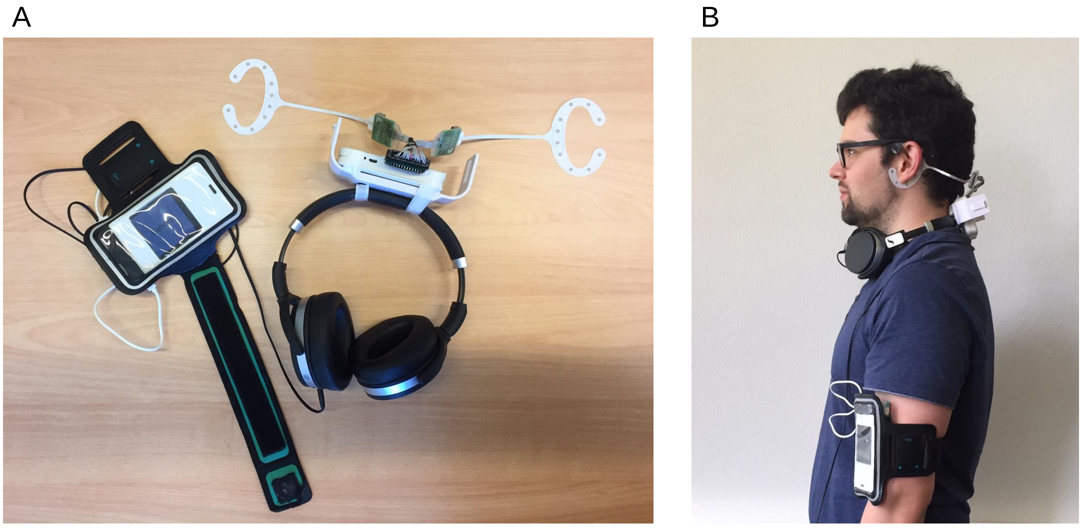

Supplementary Figure 1. Complete experimental setup. **(A)** cEEGrids were connected to the amplifier, the amplifier was taped to the headphones, and the headphones were connected to the smartphone via an audio cable. A power bank (not visible) was connected to the smartphone and both were stored in an arm-pouch. The smartphone both recorded data and presented the sounds. **(B)** Participant wearing the setup. The headphones were worn around the neck and the smartphone was attached to the arm. The depicted person provided written consent to use this photograph.

DOI: http://dx.doi.org/10.3758/s13428-021-01538-0

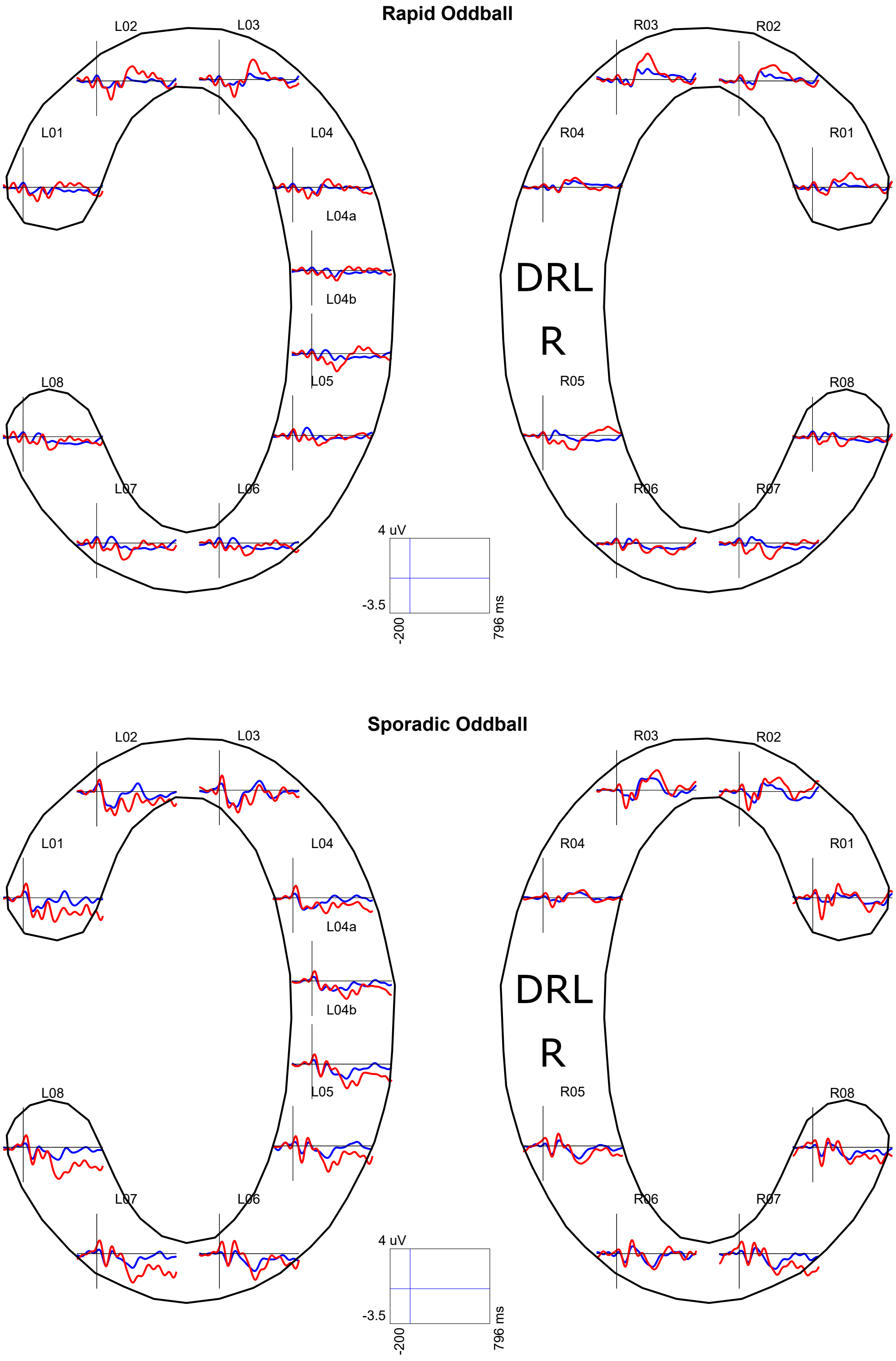

Supplementary Figure 2. Grand average of channel ERPs shown in the cEEGrid layout including electrode labels. Standard tones are blue and target tones are red. DRL: Driven-right-leg; R: Reference electrode. Top: Grand average ERPs from the rapid oddball. Bottom: Grand averaged ERPs from the sporadic oddball.

DOI: `http://dx.doi.org/10.3758/s13428-021-01538-0`

Supplementary Table 1
GLMMs for reaction times

Fixed Effects	Model Summary			
	ß	SE	t-value	p-value
Intercept	0.91	0.06	14.40	<.001
Oddball Variant	-0.39	0.05	-8.08	<.001

Random Effects			
	Name	Standard Deviation	Correlation
Participant	Intercept	0.07	
	Slope	0.07	-0.80

Note: GLMM was fitted with an inverse Gaussian distribution and an inverse link. Coefficients, standard errors, and standard deviations are not back-transformed. The intercept is the predicted mean reaction time in $seconds^{-1}$ for the rapid oddball; the beta for oddball variant represents the predicted mean change (on the link scale!) when moving from the rapid to the sporadic oddball.

Supplementary Table 2
Model comparison for reaction times

	AIC	BIC	log-likelihood	χ^2	*df*	*p*
F: Gaussian, L: Identity	4088.9	4121.8	-2038.5			
F: Gaussian, L: Log	4081.5	4114.4	-2034.8	0	0	ns
F: Gaussian, L: Inverse	4080.1	4112.9	-2034.0	0	0	ns
F: Gamma, L: Identity	1290.4	1323.2	-639.2	0	0	ns
F: Gamma, L: Log	1287.0	1319.8	-637.5	0	0	ns
F: Gamma, L: Inverse	1285.1	1317.9	-636.5	0	0	ns
F: Inverse Gaussian, L: Identity	700.9	733.7	-344.43	3388.1	0	<.001
F: Inverse Gaussian, L: Log	697.3	730.2	-342.7	3384.2	0	<.001
F: Inverse Gaussian, L: Inverse	695.1	728.0	-341.6	3385.0	0	<.001

Note: F = Family; L = Link function; ns = non-significant

DOI: http://dx.doi.org/10.3758/s13428-021-01538-0

Supplementary Table 3
GLMM for response accuracy

Fixed Effects	Model Summary			
	ß	SE	z-value	p-value
Intercept	-3.31	0.43	-7.61	<.001
Oddball Variant	1.15	0.39	2.99	<.01

Random Effects			
	Name	Standard Deviation	Correlation
Participant	Intercept	1.10	
	Slope	0.88	-0.88

Note: GLMM was fitted with a binomial distribution and a logit link function. Coefficients, standard errors, and standard deviations are not back-transformed and represent the predicted log odds of *missing* a target. The intercept is the predicted mean log odds of missing for the rapid oddball; the beta for oddball variant is the predicted mean change in log odds when moving from the rapid to the sporadic oddball.

DOI: http://dx.doi.org/10.3758/s13428-021-01538-0

Supplementary Table 4
LMM for P3 amplitudes rapid oddball

Fixed Effects	Model Summary			
	ß	SE	t-value	p-value
Intercept	1.07	0.22	4.84	<.01
Type of tone	1.75	0.31	5.56	<.001

Random Effects		
	Name	Standard Deviation
Participant	Intercept	0.51
	Slope	0.59

Note: The intercept is the predicted mean ERP amplitude (μV) for the standard tone. The beta for type of tone is the predicted mean change in ERP amplitude when moving from the standard tone to the target tone (i.e., the difference in amplitude between the tones).

Supplementary Table 5
LMM for P3 amplitudes sporadic oddball

Fixed Effects	Model Summary			
	ß	SE	t-value	p-value
Intercept	1.63	0.27	5.93	<.001
Type of tone	1.20	0.33	3.64	<.01

Random Effects		
	Name	Standard Deviation
Participant	Intercept	0.62
	Slope	0.32

Note: The intercept is the predicted mean ERP amplitude (μV) for the standard tone. The beta for type of tone is the predicted mean change in ERP amplitude when moving from the standard tone to the target tone (i.e., the difference in amplitude between the tones).

DOI: http://dx.doi.org/10.3758/s13428-021-01538-0

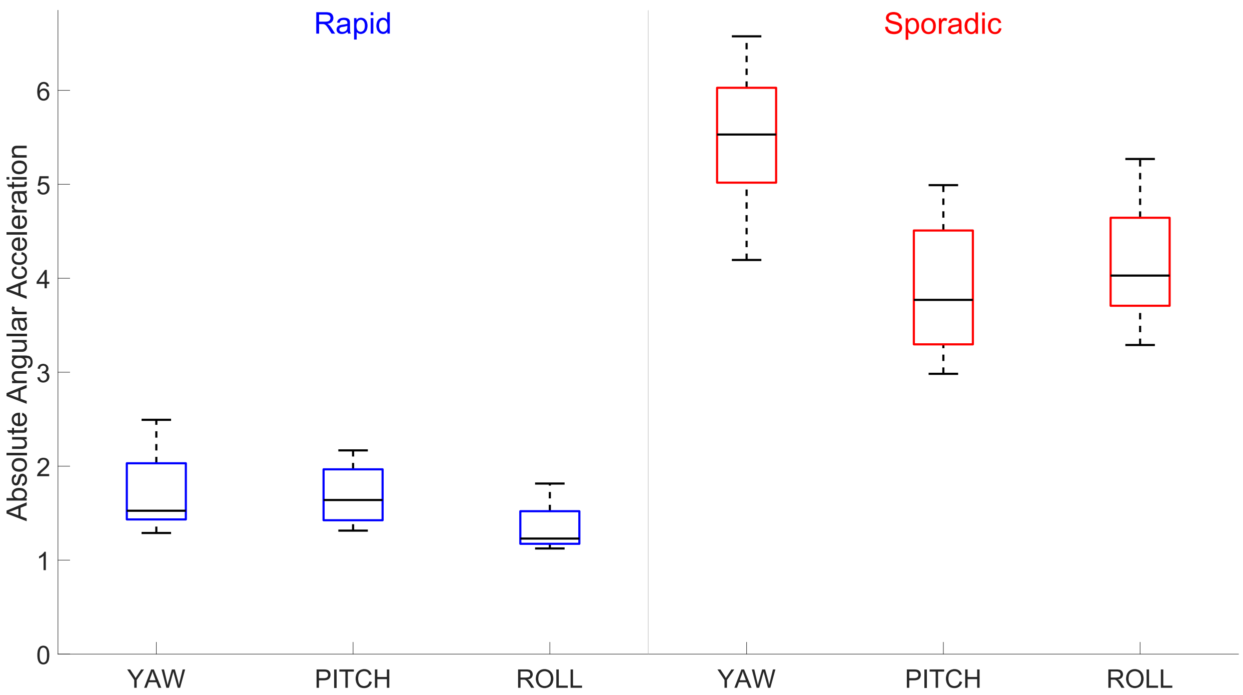

Supplementary Figure 3. Absolute values of angular acceleration per condition and gyrospcope axis. The central black line indicates the median, the bottom and top edges indicate the 25th and 75th percentile. Whiskers indicate the most extreme data points not considered outliers, which, in turn, are indicated by a black dot. Outliers were defined as data points deviating more than 1.5 times the interquartile range from the bottom or top edges.

Note on statistics: These data were submitted to a two-way repeated measures ANOVA with the factors *condition* (rapid/sporadic) and *axis* (yaw, pitch, roll). A significant main effect of condition, $F(1,7) = 125.95$, $p < .001$, $\eta^2 = .88$, and axis, $F(2,14) = 38.76$, $p < .001$, $\eta^2 = .35$, was found. The interaction was also significant, $F(2,14) = 49.47$, $p < .001$, $\eta^2_G = .27$. Post-hoc tests revealed that in the sporadic condition, both pitch and roll significantly differed from yaw ($p < .01$). Furthermore, the corresponding axes from both conditions also differed significantly from each other (all $p < .001$).

DOI: http://dx.doi.org/10.3758/s13428-021-01538-0
